# Development of Combinational Circuits by Encoding on the Basis of Developmental Biology

**DOI:** 10.1155/2020/7696398

**Published:** 2020-06-29

**Authors:** S. B. Sivasubramaniyan, R. Seshasayanan, N. Ramadass

**Affiliations:** ^1^Department of Electrical and Electronics Engineering, Meenakshi Sundararajan Engineering College, Anna University, Chennai, India; ^2^Department of Electronics and Communication Engineering, College of Engineering, Guindy, Anna University, Chennai, India

## Abstract

The present work visualizes the evolution of primitive digital circuits as a development problem. The development of the digital circuit is implemented similar to the development of a human embryo from a single cell to the complete organism. The constituent parts making up a primitive digital circuit are encoded into binary strings. Each binary string is viewed as a cell, and several such cells are allowed to adhere and multiply before culminating into a developed organism. The binary string of the cell is further mapped to a particular attribute which defines the constituent of the complete digital circuit implemented. The present work illustrates the development of a 4-input combinational digital circuit. The development of 2-input majority function is illustrated, and the results are shown for the 2-input Ex-OR gate, 2-input majority function with 4 input variables, and a 2-to-1 multiplexer circuit. The development of the digital circuit resembles the development of an embryo in a living organism.

## 1. Introduction

The biological world has demonstrated that the fittest organism survives over time. A change in the organism's genome results in initial changes in the phenotype. These changes are manifested in the offspring over time. We refer to this process as evolution. The change occurring in the organism's genotype is either random or forced by the environment. Understanding and mimicking nature has led to a whole new field of evolutionary algorithms, whose primary objectives are search and optimization of multivariable problems. Genetic algorithm (GA), evolutionary strategies (ES), and evolutionary programming (EP) are alternative strategies followed by different groups of researchers working on a range of diverse problems.

The present work is based on the premise that development is the key to evolution. The adaptability of the any organism rests on the flexibility of the organism to the changes within and outside it. The flexibility and the eventual adaptability would be meaningful only when the organizational framework of the organism is intact. Thus, development is seen as the key for evolution of a species. The proposed work is an attempt to emulate the adaptability of the biological organism to digital hardware.

Biological species undergoes a highly deterministic developmental cycle. The embryonic development of any species starts with a single cellular organism. The single cell undergoes process consisting of more than one developmental stage, crafted naturally, to become a multicellular organism. The proposed work is aimed at creating a developmental cycle for the design of digital hardware. Digital hardware is represented by a Boolean expression. A Boolean expression consisting of few gates can be represented either as Sum of Product or as Product of Sum expressions. The proposed work tried to find out whether the Boolean expression can be developed using primitives mimicking the developmental cycle of a biological organism.

The present work is also motivated by attempts including Mandelbrot sets, in which Mandelbrot developed patterns using a simple equation. Lindenmayer systems, commonly referred as L-systems, relied on step-by-step procedures to develop complex and symmetric patterns from single primitives.

The present work proposes a developmental algorithm, similar in line with the embryonic development of a biological species, for the electronic circuit. In the process, distinct encoding of each component of a circuit by a binary string is carried out. Encoding of each of the component and its association with other components of the circuit is carried out which resembled the DNA encoding of the biological species. The binary representation also paved the necessary edge to include mutation by means of flipping of bits. The genome of a circuit is considered to be specific to the functionality of the circuit.

The proposed work aims at the development of the electronic circuit as an embryo develops in a biological world. This is carried out by binary encoding of each of the components and then designing an algorithm for the development of the circuit. The proposed work would be an ideal platform to the design of fault tolerant systems and self-healing circuits.

## 2. Literature Survey

There have been attempts carried out to determine the adaptability of various evolutionary methods in optimization of various problems [1]. Among the engineering problems successfully adopted for the practical hardware design, the most important are the design of an antenna [2], table [3], rotor blade design [4], and physical properties of the silicon substrate [5]. It may be mentioned in passing that social behavior of birds [6], lion [7–9], and human beings [10] also has been formulated as an optimization problem.

The development of a digital circuit with fundamental gates starts with a truth table of the circuit. For *n* variables, it requires 2*n* combinations to be checked for determining a Boolean expression. This problem is referred to as the satisfiability (SAT) problem. The time required for a Boolean SAT problem increases exponentially with increase in the number of variables. Perdrycz et al. handled the challenges in applying evolutionary computation (EC) for a Boolean SAT problem by converting the Boolean problem to a continuous domain [11]. De Jong and Spears handled the same by converting Boolean variables to floating point numbers [12]. Slowik and Bialko in [13] presented a comprehensive survey on the application of EAs to digital circuit design.

The idea of evolving optimized circuits by encoding the components and the interconnections among them has been demonstrated in [14]. An attempt to apply genetic programming to evolve a fit computer program is presented by Koza in [15]. Coello [16] employed the genetic algorithm to design adders and multipliers. In [17], Miller et al. proposed genetic algorithm-based combinational circuit design which was modeled on a FPGA. These efforts led to the design and development of digital circuits built on an array of gates controlled by predetermined criteria [18, 19]. A modified model, called the development model having two layers, a protein layer and an architecture layer, was proposed by Gordon and Bentley in [20]. The present work aims at developing a digital circuit without a predefined array. The later sections discuss the same in detail.  Section 2 deals with the system design using artificial cells. The proposed algorithm is given in Section 3. A combinational circuit example is discussed in Section 4.  Section 5 discusses the results of the algorithm.

## 3. System Design with Artificial Cells

### 3.1. Development of Combinational Circuits

This paper proposes a model for the development of a digital circuit. Various components making up the circuit are visualized as a biological cell making up an organ. Each component, with its associated input pattern, is encoded with a unique binary string. For a 7-level digital circuit, the length of the encoded binary string for a single cell is defined by an empirical relation given in the following equation:

(1) $$\begin{array}{c} \text{length of the genome}=\log_{2}i+2i+13\ldots \ldots \ldots , \end{array}$$

where “*i*” is the number of inputs to the logic circuit. The genetic strip is referred to as the genome of the cell, and the combination of several cells makes up the eventual digital circuit. The visualized genetic strip accommodates designs consisting of basic logic gates, derived gates, and universal gates along with logic circuits. The unused genes merely serve the purpose of filling up spaces as of now.

A Sum of Product (SoP) expression is taken for the illustration. The truth table of the problem is taken as an input. This makes the problem a Boolean satisfiability (SAT) problem. The time required to attain the solution increases with the number of variables being taken. The major challenge to the SAT problem is the definition of the fitness function which is problem-dependent. The natural way is defining the fitness function with logical “1” entries in the truth table. However, it is understood that logical “1” is not the complete solution of the function, rather one of the possibilities of the complete solution, as all the functions making up the truth table are equally competitive [11]. This is justified as the development problem overcomes the scalability issue associated with the digital circuit evolution [20].

The proposed idea contains problem-specific (species-specific) cycles and each cycle comprising one or many phases. The cycles and phases are modeled to follow the steps presented in the following.

#### 3.1.1. Birth of Zygote

Artificial cell division starts with an initial randomly chosen single cell made up of the binary string. The single cell represents the zygote in our artificial cellular development. Naturally, one of the functions in the truth table having a logic “1” value will be the zygote. The zygote is randomly chosen from all the possibilities posed by the truth table of the function. If the zygote fails the fitness test, it eventually ‘dies,' and the new zygote is produced. The birth of zygote is referred as phase I of the artificial model.

#### 3.1.2. Cell Division: SAT Problem

In phase II, the single cell divides itself into two. The two daughter cells will have a single-bit change from the parent. Again, each of the daughter cells divides into two. The single-bit change will take place in one of the frames denoting the input type. Cells divide till the “telomere index” becomes zero. The telomere index is defined in the later sections.

#### 3.1.3. Single-Bit Change: Concentration Gradient and the Inducing Factor

**Figure 1:**
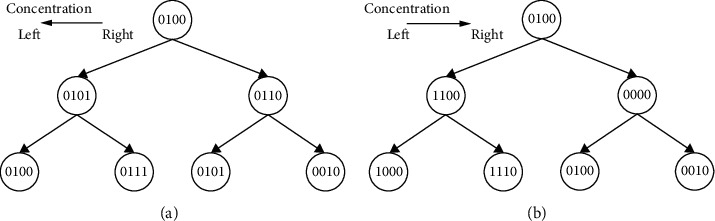
Parent and daughter cells differing by one-bit change.

In natural cells, the concentration gradient and the inducing factor start from one point and gradually reach some other point of the cell during gastrulation. The single-bit change resembles the concentration gradient in the presence of the inducing factor in the biological world. If the bit string is “0100,” the daughter cells produced would be “0101” and “0111.” This is after considering that the bit change is taken from right to left. If it is taken from left to right, it would be “1100” and “1000.” Figure 1 shows the production of daughter cells from parent cells. The natural processes stated above—cell division, blastulation, and gastrulation—are assumed to take place simultaneously in our artificial model. We refer this as phase II. The single-bit changes in the strings are checked for their fitness. If the cells are not fit, the telomere index is unaltered. If the cells are fit and there is no repetition, the telomere index is decremented by one. The fitness test carried out is the genotypic fitness.

At the end of gastrulation, morphogenesis takes place. Morphogenesis defines the structure to the organism. Several types of morphogenetic processes are described [21]. The morphogenetic processes are species-specific and organ-specific in a particular species. Similar in line with that, the proposed morphogenetic process is designed to follow condensation in which mesenchymal cells undergo local cell division and consequent cell adhesion. Slack describes that the cells having similar cadherins adhere than the cells with dissimilar cadherins [21].

#### 3.1.4. Local Cell Division: Cell Replication

This is similar to the local cell division in the biological world. Cells which undergo adhesion are defined by a factor denoted as “specificity factor” or simply “s-factor.” Adhesion is defined for the cell pair which has a high specificity factor. If the two cells have the exact genetic material, they fuse together to form one cell, so s-factor of identical cells is discarded. If two cells have only one-bit (gene) change between them, then the cells are said to have more s-factor. Gate-dominated adhesions are possible only for cells having one-bit difference among them. Gate-dominated adhesions are referred as “gadherins.” Specificity factor (s-factor) also plays a role in local cell division or cell replication. A replication index or “*r*-index” (or simply “*r*”) is defined for the number of identical copies the cell makes up during local cell division. s-factor and *r*-index work sequentially. Each cell having high “s-factor” is given (*r* − 1) replications in the local cell division.

#### 3.1.5. Cell Adhesion and Specificity: Laws of Complementarity

Before any cell adhesion is possible, cells are tested for their specificity with each other cell. A “*n*-”cell system will have *n*C_2_ number of combinations possible. Say, for example, a 4-cell system will have 6 possible combinations of two cells taken at a time (a 4C_2_ problem). All the resultant combinations are tested for their specificity. Cells with high s-factor now are chosen to take part in gate-dominated adhesions after cells locally make (*r* − 1) copies of it.

**Figure 2:**
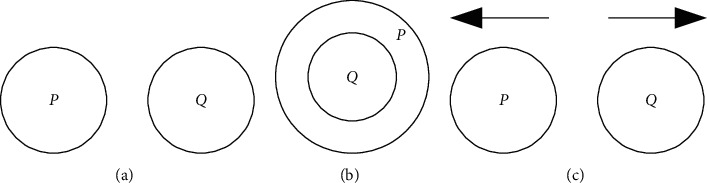
Two cells adhere together if the s-factor is high or move apart if the s-factor is low. (a) Two cells. (b) Adhesion of two cells (s-factor being high). (c) Two cells move apart (s-factor being low).

Each copy of the cell undergoes adhesion with the other types of the cell. This takes into account the Boolean function, *X* + *X*′ = 1 and X · X′ = 0. The cell adhesion produces distinct cells after the fusion; the cells which have high specificity factor alone produce the fused products. The cells which do not find a proper ally to adhere stay in the system unscathed as shown in Figure 2.

There are two types of cell fusion defined in the artificial world—AND fusion and OR fusion. The SoP realization, obviously, follows AND fusion in cycle I of development. The prerequisite for two cells to undergo fusion (adhesion) is that the two cells must have only one-bit difference between them. If the prerequisite is met, the cells undergo adhesion and produce a single distinct cell with a logic ‘0' in the allele which had the single-bit difference. OR fusion makes a logic “1” in the corresponding allele. We refer this as gate-Dominated adhesion or simply, gadherins. AND fusion is referred as gadherin type-1 or GT-1 adhesion and OR, GT-2 adhesion.

#### 3.1.6. Differential Strength of Adhesion: Consensus Theorem

The strength of adhesion of each cell with the other cell is quantified to minimize the logic function being implemented. The differential strength of adhesion also determines the stay of the cell in the system. Each cell adhesion forms “1” (not a logical “1”). If the number of cells forming “1” is two in number, the two cells contribute equally to the adhesion—0.5 each, 0.25 each if there are 4 cells, and so on. If the same cell also forms the adhesion product with another cell, the cell further loses half of its strength—the initial 0.5 becoming 0.25, and so on. This makes the original tally of “1” to reduce.

**Figure 3:**
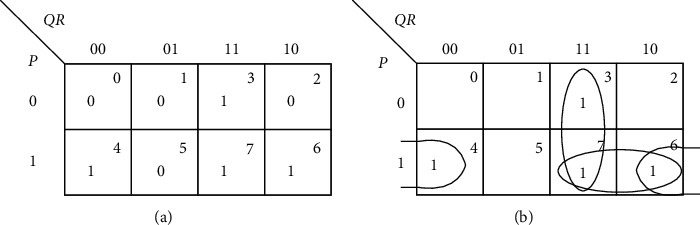
Grouping of 3-input function. (a) [3, 4, 6, 7]. (b) Group (3, 7), (6, 7), (4, 6).

The reduction is permitted as long as the strength is not less than the individual contribution; in that case, the particular cell adhesion is ruled out. This takes into account the consensus theorem of the Boolean algebra.  Figure 3 shows a 3-input function.  Table 1 explains the differential strength of adhesion. The initial values are the values each cell possesses and final is the value each cell acquires at the end of all possible adhesions. When the cells adhere, the individual strengths of the cells account for the group.

Table 2 indicates the removal of weaker cells from the group. Table 2 signifies the phenotype fitness.

Phases I to III form cycle I in the proposed model. Cycle II follows cycle I based on the constraints given in the problem in hand. The presence of further cycles also depends on the problem.

### 3.2. Fitness Function

Inputs are fed as the constraint to be satisfied for the problem. The function that produces logic “1” is checked with all the combinations of the given set of inputs. These combinations are individually referred to as parts of the circuit to be formed. The fitness function is problem specific and more importantly, each cycle could well be assumed be have different fitness function either for components or for the nature of the inputs.

### 3.3. Illustration of the Genome

The circuit consists of various parts (or cells). Each cell is represented by a binary string referred as the genetic strip, genome, or chromosome. Each genetic strip is decoded into 5 frames. The length of each genetic strip or chromosome is determined using equation (1). Frames are named as A, B, C, D, and E. Each genetic strip (chromosome) is unique for a particular cell during its birth. Upon development, the chromosome of a particular cell evolves and during the process, becomes robust. The cell achieves robustness by adhesion with the other cell. The number of bits or genes of the chromosome remains the same before and after the adhesion. The change gets affected only in the content of the chromosome, only at the particular allele, where the fusion takes place. The development starts with a single cell. The single cell is allowed to undergo the fitness test. After testing for fitness, the cell divides producing two daughter cells which in turn produce 4 daughter cells, and so on.

A genetic strip or a chromosome with five frames is shown in the following. There is a start bit “1” before frame A and a stop bit “1” after frame E. The start and stop bits in the genetic strip represent the telomeres in the biological environment [21]. However, this electronic counterpart differs in two respects. The natural telomeres have repeated sequences of nucleotides, whereas in the proposed work, it is taken to be a single bit at the first and the last position of the genetic strip. The second difference is that the natural telomeres tend to lose few nucleotides during every time the cell divides, whereas in the defined telomere, it is taken care by the telomere index. The telomere index gets decremented each time the cell divides. By the time the telomeres fully disappear, the cells stop dividing. The artificial telomeres mere serve the purpose of start and stop bits of the genetic strip.


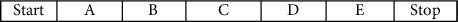


Each frame denotes an attribute of the chromosome. The details are shown in the following.


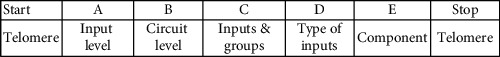


Equation (1) governs the size of the genome. For a 4-input and 7-level circuits, the size of the genome is as shown in the following. Frames C and D depend on the number of inputs as shown.


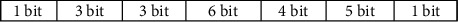


The idea and the roles played by frames A and B are presented in Table 3.

Frame A in the strip indicates the level from which the input is applied to the circuit. Frame B represents the level of the circuit being considered.  Table 4 explains the encodings of frames C and D. Frame C is made up of two smaller frames—c1 and c2. For a 4-input system considered, c1 has 2 bits, and c2 has 4 bits. Frame D represents whether the considered inputs are taken straight or complemented.

Table 5 denotes the encodings of frame E. Frame E has 5 bits with first 2 bits representing the component index, and the last 3 bits represent the type of the component.

Also, frames C and D undergo layered mutation. It is defined as one wherein a bit-level (gene) change in one frame affects the other. The change in frame D affects a corresponding change in frame C. The corresponding changes in frames C and D are checked for a specific combination of bits (genes) in frame A, B, and E.

Once this is checked and deemed fit, the other set of genes is set in frames A, B, and E, and frames C and D are allowed to change. Layered mutations get affected in all the variations that happen in frames C and D.

A chromosome given as 10010011111101011 represents a 2-input AND gate. It is decoded as shown in the table which is given in the following.


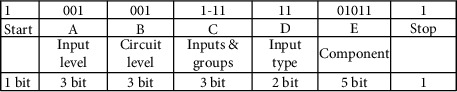


The chromosome has 18 genes arranged in 5 frames. Frame A has 001. This refers to the primary inputs which are fed to the prescribed gate (to be decoded with frame E). Frame B refers to level I of the circuit. Frame C has two subframes—c1 and c2. “1” in c1 refers that the number of inputs is 2 (say, P and Q), and “11” in c2 denotes that both inputs are considered. Frame D which is decoded for the type (whether considered straight or complemented) of inputs denotes that the 2 inputs (P and Q) are taken straight without complementing. Frame E has 5 bits. The first 2 bits are 01 which refers to Index 1 of the component. Component Index 1 refers to the fundamental gates—basic (AND, OR, and NOT), universal (NAND and NOR), and derived (Ex-OR and Ex-NOR). Frame E in the given genetic strip refers to AND gate (011). There is 1 bit each to signify the start and stop of the genetic strip. Overall, the chromosome referred by 10010011111101011 is decoded into *P*·*Q*, or in other words, input *P* and input *Q* are fed to 2-input AND gate.

With frame E as (01010) instead of (01011) would make the component to be an OR gate. This makes the function realized into *P* + *Q*. If the change is affected in frame D with 01 in the original genome, the function realized is *P* · *Q*′ instead of *P* · *Q*.

### 3.4. The Frame Swing

As stated already, each frame in the genetic strip has different fitness functions. The fitness check of the individual frames is carried out at different scales. The string change in frames A, B, and E occurs in parallel. Frame C undergoes random variations and completes its full set for a single variation with frames A, B, and E. Frame D undergoes all possible changes for each string change in frame C. We refer this as the frame swing (Algorithm 1).

### 3.5. The Proposed Algorithm

### 3.6. Mapping

The proposed design is aimed at generating the net-list of the design. The genetic strip given in the example—100100111011011010111—producing *P* · *Q*—maps the interpreted function into a net-list as *Y* ≤ *P* and *Q*. The net-list generated could be validated using a reconfigurable device, and the same could be fed to the back end tool for implementation. The development time taken by a design is determined by the complexity of the circuit being developed in analogous to the biological world where embryonic development among different species differs considerably.

## 4. Development of Combinational Circuits

### 4.1. Two-Input Majority Function

An example of input majority function is taken to illustrate the development. The truth table of a two-input majority function is shown in Table 6.

### 4.2. Length of the Genetic Strip

The length of the strip is defined from equation (1) which makes it 21 bits.

### 4.3. Cycle I

The inputs considered are level I (primary) inputs. The circuit level considered is level I as in the case of any Boolean SAT problem.


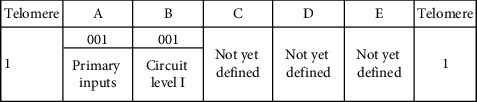


The problem in hand dictates the choice of gates chosen initially. Since the problem is a Boolean SAT problem, the chosen gate is an AND gate.


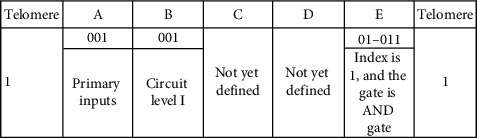


### 4.4. Constraints

Constraint 1—number of inputs = 3; constraint 2—final cell population = 4; constraint 3—minterms in the truth table which have logic “1” = [3 5 6 7].


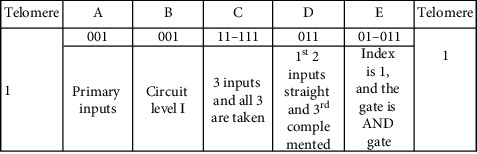


### 4.5. Phase I: Fertilization

Thus, the fertilized zygote is given by 100100111111011010111. The genetic strip of the chromosome is tested for its fitness. The content of the D register is tested for fitness. Since 011 has logical “1” in the truth table, it is tested fit.

### 4.6. Phase II: Cell Division: Embryogenesis—Genotypic Fitness

**Figure 4:**
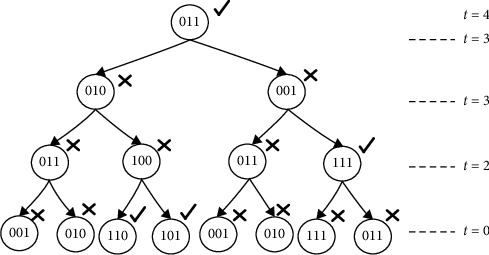
Cell division for a 2-input majority function with 3 input variables.

The telomere index and the final products of the cell division are shown in Figure 4. Each parent cell produces two daughter cells which differ from their parent cell by one bit. The inducing factor is taken from right to left. Only the contents of the D register are shown in Figure 4.

The final cell population is given by *n* = 4.

### 4.7. Phase III: Cell Adhesion—Morphogenesis

There are 4 cells at the end of phase II as given in Table 7; all the 4 cells are tested for their specificity factor (s-factor) with all the other cells. Thus, 4 cells after *n*C_2_ result in 6 combinations. The higher s-factor dictates the combinations of cells which adhere themselves.  Table 8 shows the cells and their s-factor.

The sorted combinations of cells based on s-factor are shown in Table 9.

The *r*-index or the replication index tells how much copies each cell in the combinations makes. For this to happen, the combination must have higher (≥0.95) s-factor. Each cell is given (*r* − 1) copies.  Table 10 shows the replication index (*r*-index) of cells after adhesion.

### 4.8. Local Cell Division

**Figure 5:**
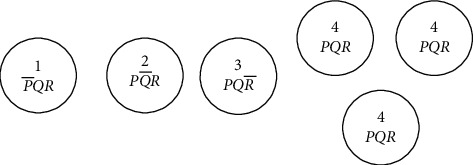
Local cell division.

Each cell makes (*r* − 1) copies of it. This is shown in Figure 5.

**Figure 6:**
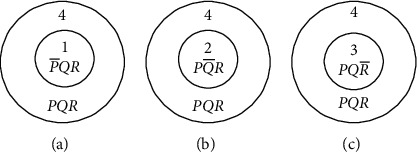
Cell adhesion.

Each cell adheres to the other cell types. Cells 1–3 combine with the three copies of cell 4 as shown in Figure 6. Gate-dominated adhesions, gadhrines (gadherin type 1—GT1), are given in Table 11.

### 4.9. Layered Mutation

Layered mutation will affect a corresponding change in the C frame, i.e., a second bit change in the D frame due to cell adhesion will cause a corresponding second bit change in the C frame.

The new fused cell has a bit changed to logic ‘0' in frame C corresponding to the allele which is fused (adhered) in frame D.  Table 12 shows the layered mutation.

### 4.10. Cell Adhesions

There are 3 cells at the end of first local cell division, and all the 3 cells are tested for their specificity factor (s-factor) with all the other cells as given in Table 13. Thus, 3 cells after *n*C_2_ result in 3 combinations.

The higher s-factor (≤0.95) dictates the combinations of cells which adhere themselves.

No combination has high s-factor. Hence, cells have *r*-index as numerical 0, which means no replication and no further adhesion. All the three cells are distinct as shown in Table 14.

### 4.11. Differential Strength of Adhesion

The differential strength of adhesion between cells is determined to find out the redundant cells forming the organism. The contents of frames C and D determine the differential strength of adhesion.

### 4.12. Determination of the Number of 1s Forming Each Cell

The last column of Table15indicates the number of 1 s forming the cell. The difference between the number of inputs and the number of inputs grouped eventually is used to determine the number of 1s in a cell.

**Figure 7:**
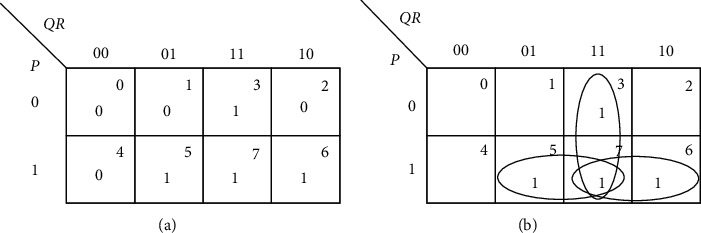
Differential strength of adhesion. (a) [3, 5, 6, 7]. (b) Group (3, 7), (6, 7), (5, 7).

Table 16 shows that there are two 1s in each of the cells. The last column in Table 16 indicates the number of 1s. All the cells—*QR*, *PR*, and *PQ*—look identical as far as the number of 1s making up the group is considered.  Figure 7 testifies Table 16 with the help of a Karnaugh map.

The Ex-OR operation of the D frame and c2 subframe results in 0s. This indicates that there are no changes already done to the D frame as given in Table 17. The presence of logical 0 after the Ex-OR of the c2 subframe and D frame indicates that 0s in the product can be toggled to 1s to determine the constituents (implicants) of the cells. In contrast, the presence of logical 1 after the Ex-OR of the c2 subframe and D frame indicates that 0s in the corresponding allele should not be toggled to find out the constituents (implicants) of the cell.

### 4.13. Removal of Weaker Cells Having Lower Strengths of Adhesion: Phenotypic Fitness

To remove the weaker constituent from the group of cells, the individual strength of each of the constituent is checked. Section 3 describes in detail the idea behind the differential strength of adhesion. Each of the constituent in the present example takes a “strength” of 0.5 since there are two constituents in each cell.  Table 18 shows the individual “strength.”

Each adhesion makes the individual constituent to lose half of its strength to the fellow constituent. In Table 19, constituent 7 in the cell (3, 7) has a “strength” of 0.5 after adhesion 1. The same constituent loses half of its “strength” and becomes 0.25 as it undergoes one more adhesion. The individual constituent or implicant can survive to make as many adhesions as possible with the condition that the combined strength of adhesion of the individual cell should not fall below the initial strength as given in Table 18.

All the three cells do have higher strength of adhesion, hence forms the cells in cycle I of development as shown in Table 20.

### 4.14. Cycle II: Cell Grouping

Cycle II of the development has minor changes from the previous cycle. Input level and circuit level are updated in frames A and B, respectively. Frames C and D have the inputs decoded and are taken straight. The outputs of cycle I form the inputs for cycle II. OR gate forms the component of interest in cycle II as the problem in hand is a Boolean SAT problem. The number of inputs is 3 (more than 2 and odd) at the end of cycle I. The 3 inputs are grouped into 2, and the output of the OR gate is further made to undergo logical OR operation with the third input to ensure two-input gate implementation. The circuit level is interpreted as the same for all the three inputs, i.e., the 3-input OR gate is made up of two 2-input OR gates.

The following cells constitute the 2-input majority function:

  100100111110110010111
  100100111101101010111
  100100111011011010111
  101101111111111010101

**Figure 8:**
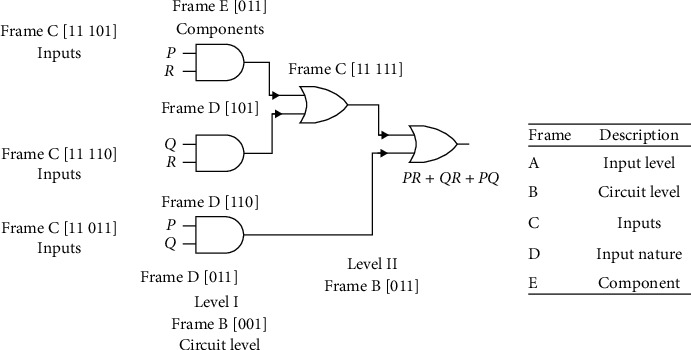
Implementation of 2-input majority function with 3 inputs.

The same can be interpreted as follows. Decoding a 2-input majority function with 3 inputs results in a genetic strip of 4 × 21 bits given by 10010011111011001011110010011110110101011110010011101101101011101101111111111010101.  Figure 8 shows the implementation of 2-input majority function with each frame explicitly shown.

## 5. Developmental Results

Application of the proposed algorithm to three primitive combinatorial circuits gave the following results as listed in Table 21. The length of the genome increases with the increase in the number of inputs. The total number of inputs determines the cell length, and the number of logical 1s in the truth table determines the number of cells which in turn determines the length of the entire genome.

Table 21, the length of the cell and the eventual length of the genome depend on the type of the circuit being solved. This is analogous to the biological world in which each species' genome is unique. However, there are certain similarities in the genome of various species. This is true to the genome of different circuits as well as illustrated earlier.

## 6. Conclusion

The development of a combinational digital circuit based on developmental biology is carried out for 4 input circuits. The development of a digital cirucit from a single cell is seen to follow similar steps as in the natural world. The development is conceptualized around a 23 bit binary string for the primitive 4 input circuits. The length of the genome increases with the increase in the number of input bit width. For a circuit with a maximum of 8 bit input, the length of the genetic strip is 32 bits. For a 16 bit input, the length of the genome will be 49 bit length. The development of combinational circuits can be extended up to 7 levels with the empirical relationship presented in this paper. For bits of higher order, say 128 bits, the length of the genome increases proportionately. For instance, the length of a single cell would be 155 bits. The development of higher-order strings would be taken up as the future work. As an illustration, primitive combinational circuits such as 2-input Ex-OR gate, 2-input majority function for a 3-input circuit, and a 2-to-1 multiplexer circuit are taken up for the developmental algorithm developed. The examples show how the circuit develops in the presence of redundant terms. The future work would consider implanting the same with a sophisticated tool to perform the generation and mapping of the particular evolved net-lists. However, the present work ends with mapping of the strips to the specified components using the simulation tool.

## Figures and Tables

**Algorithm 1:**
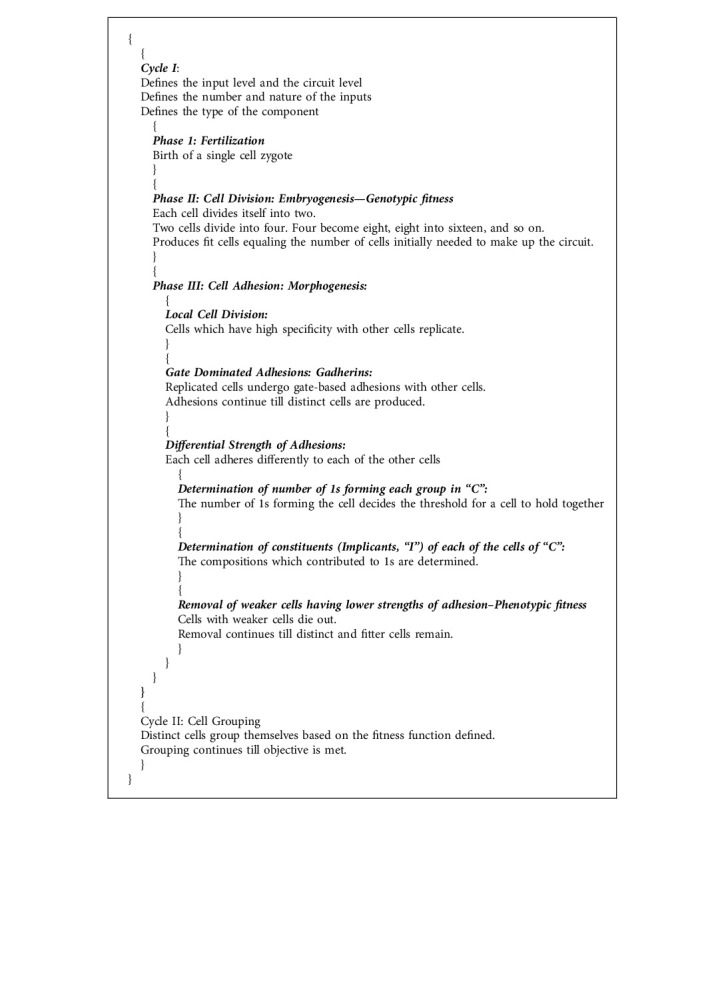
Womb for combinatorial circuits development.

**Table 1:** Differential strengths of adhesion for the map shown in Figure 3.

Strengths	*f*	3	4	6	7
Initial		1	1	1	1
(3, 7)	2	0.5	—	—	0.5
(6, 7)	2	—	—	0.5	0.25
(4, 6)	2	—	0.5	0.25	-
Final		0.5	0.5	0.25	0.25

**Table 2:** Removal of weaker cells.

Adhered cell	Combined strength	Total	Status
(3, 7)	0.5 + 0.25	0.75	Selected
(6, 7)	0.25 + 0.25	0.50	Discarded
(4, 6)	0.5 + 0.25	0.75	Selected

**Table 3:** Encoding of frames A and B.

String	Frame A (input level)	Frame B (circuit level)
001	Level I	Level I
011	Level 2	Level 2
010	Level 3	Level 3
110	Level 4	Level 4
111	Level 5	Level 5
101	Level 6	Level 6
100	Level 7	Level 7

**Table 4:** Encoding of frames C andD.

Frame C (number of inputs and input groups)							Frame D (type of inputs)
Inputs, *i* c1					Input groups, c2		c-complements-straight
*I*	*w*	String	*I*	*w*	Group	String	String
1	—	—	—	—	—	—	—

							00 – c, c
							01 – c, s
2	2	1	2	2	2 at a time	11	10 – s, c
							11 – s, s

							000 – -, c, c
							001 – -, c, s
					2 at a time	011	011 – -, s, s
							010 – -, s, c
						101	000 – c, -, c

3	2	11	3	3			
						110	.
					3 at a time	111	.

4	2	10	4	4	2 at a time	0011	.
						0101	.
						.	.
						1100	.
					3 at a time	0111	.
						1011	.
						1101	.
						1110	.
					4 at a time	1111	.

5	3	110	5	5	2 at a time	00011	.
						.	.
					.		.
					5 at a time	11111	.

**Table 5:** Encoding of frame E.

Frame E (components)		
Index (encoding)	Index (decoded)	String
00	—	—

01 (gates)	Not defined	000
	Ex-OR	001
	AND	011
	OR	010
	Ex-NOR	110
	Buffer	111
	NOR	101
	NAND	100

11 (circuits)	Mux	000
	Demux	001
	Decoder	011
	Encoder	010
	.	110
	.	111
	.	101
	.	100

10	Empty	Empty

**Table 6:** Truth table of 2-input majority function.

*P*	*Q*	*R*	*Z*
0	0	0	0
0	0	1	0
0	1	0	0
0	1	1	1
1	0	0	0
1	0	1	1
1	1	0	1
1	1	1	1

**Table 7:** Cells after cell division.

Cell number	Cells	Minterm
1	100100111111110010111	P′QR
2	100100111111101010111	PQ′R
3	100100111111011010111	PQR′
4	100100111111111010111	PQR

**Table 8:** Cells and their s-factor.

Cell combinations	Cells	Ex-OR	s-factor
1 with 2 *P′QR* with *PQ*′*R*	100100111111110010111 100100111111101010111	1001001111111**10**010111 1001001111111**01**010111	0.91
1 with 3 *P*′*QR* with *PQR*′	100100111111110010111 100100111111011010111	100100111111**1**1**0**010111 100100111111**0**1**1**010111	0.91
1 with 4 *P*′*QR* with *PQR*	100100111111110010111 100100111111111010111	10010011111111**0**010111 10010011111111**1**010111	0.96
2 with 3 *PQ*′*R* with *PQR*′	100100111111101010111 100100111111011010111	100100111111**10**1010111 100100111111**01**1010111	0.91
2 with 4 *PQ*′*R* with *PQR*	100100111111101010111 100100111111111010111	1001001111111**0**1010111 1001001111111**1**1010111	0.96
3 with 4 *PQR*′ with *PQR*	100100111111011010111 100100111111111010111	100100111111**0**11010111 100100111111**1**11010111	0.96

**Table 9:** Cells sorted based on their s-factor.

Cell combina tions	Cells	Ex-OR	s-factor
1 with 4 *P*′*QR* with *PQR*	100100111111110010111 100100111111111010111	10010011111111**0**010111 10010011111111**1**010111	0.96
2 with 4 *PQ*′*R* with *PQR*	100100111111101010111 100100111111111010111	1001001111111**0**1010111 1001001111111**1**1010111	0.96
3 with 4 *PQR*′ with *PQR*	100100111111011010111 100100111111111010111	100100111111**0**11010111 100100111111**1**11010111	0.96
1 with 2 *P*′*QR* with *PQ*′*R*	100100111111110010111 100100111111101010111	1001001111111**10**010111 1001001111111**01**010111	0.91
1 with 3 *P*′*QR* with *PQR*′	100100111111110010111 100100111111011010111	100100111111**1**1**0**010111 100100111111**0**1**1**010111	0.91
2 with 3 *PQ*′*R* with *PQR*′	100100111111101010111 100100111111011010111	100100111111**10**1010111 00100111111**01**1010111	0.91

**Table 10:** Cells and their *r*-index.

Cell adhesion	Adhesion	s-factor	*r*-index			
			1	2	3	4
1 with 4 *P*′*QR* with *PQR*	10010011111111**0**010111 10010011111111**1**010111	0.96	1	0	0	1
2 with 4 *P*′*QR* with *PQR*	1001001111111**0**1010111 1001001111111**1**1010111	0.96	0	1	0	1
3 with 4 *PQR*′ with *PQR*	100100111111**0**11010111 100100111111**1**11010111	0.96	0	0	1	1
		Total, *r*	1	1	1	3

**Table 11:** Gate-dominated adhesions.

Cell adhesion	Adhesion	Adhered cell
1 G1 4 *P*′*QR* G1 *PQR*	**100100111111110**010111 10010011111111**1010111**	**100100111111110010111**
2 G1 4 *PQ*′*R* G1 *PQR*	**10010011111110**1010111 1001001111111**11010111**	**100100111111101010111**
3 G1 4 *PQR*′ G1 *PQR*	**1001001111110**11010111 100100111111**111010111**	**100100111111011010111**

**Table 12:** Layered mutations.

Cell	Cell adhesion	Adhered cell	New adhered cell after layered mutation
1	*QR*	**100100111111110010111**	**100100111110110010111**
2	*PR*	**100100111111101010111**	**100100111101101010111**
3	*PQ*	**100100111111011010111**	**100100111011011010111**

**Table 13:** Cells and their s-factors.

Cell	Cell adhesion	Cell combinations	Ex-OR	s-factor
1	*QR*	1 with 2 QR with PR	100100111110110010111 100100111101101010111	**0.83**
2	*PR*	1 with 3 QR with PQ	100100111110110010111 100100111011011010111	**0.92**
3	*PQ*	2 with 3 PR with PQ	100100111101101010111 100100111011011010111	**0.83**

**Table 14:** Cells after cell adhesion.

Cell	Cell adhesion	Cell
1	*QR*	100100111110110010111
2	*PR*	100100111101101010111
3	*PQ*	100100111011011010111

**Table 15:** Determination of 1s in each cell.

Cell	Cell	C frame interpretation				No. of 1s in the cell, *f* = 2^c1-c2^
		c1		c2		
		Encoded	No. of inputs	String	Inputs considered	
1	*QR*	11	3	110	2	2
2	*PR*	11	3	101	2	2
3	*PQ*	11	3	011	2	2

**Table 16:** Differential strength of adhesion.

Cell	Cell	Cell	C frame		D frame
			c1	c2	
1	*QR*	100100111110110010111	11	110	110
2	*PR*	100100111101101010111	11	101	101
3	*PQ*	100100111011011010111	11	011	011

**Table 17:** Determination of the constituent of each cell.

Cell	Cell	C frame		D frame (3 inputs)	D frame interpretation			
		c1	c2		c2 XOR D	Change already	D frame	
							Encoded	Actual
1	*QR*	11	110	110	000	No bit	110	3
							111	7
2	*PR*	11	101	101	000	No bit	101	5
							111	7
3	*PQ*	11	011	011	000	No bit	011	6
							111	7

**Table 18:** Constituents (implicants) of the cell.

Cell	Threshold		Constituents
	*F*	1/*f*	
*QR*	2	0.5	(3, 7)
*PR*	2	0.5	(5, 7)
*PQ*	2	0.5	(6, 7)

**Table 19:** Determination of strengths of individual cells.

Constituents	Adhesion 1				Adhesion 2				Adhesion 3			
	3	5	6	7	3	5	6	7	3	5	6	7
*QR* (3, 7)	0.5	0	0	0.5	0.5	0	0	0.25	0.5	0	0	0.25
*PR* (5, 7)	—	—	—	—	0	0.5	0	0.25	0	0.5	0	0.125
*PQ* (6, 7)	—	—	—	—	—	—	—	—	0	0	0.5	0.125

**Table 20:** Differential strength of adhesion.

Constituents	Adhesion 3				Total	Strengths of adhesion
	3	5	6	7		
*QR* (3, 7)	0.5	0	0	0.25	0.75	Strong
*PR* (5, 7)	0	0.5	0	0.125	0.625	Strong
*PQ* (6, 7)	0	0	0.5	0.125	0.625	Strong

**Table 21:** Combinational circuits and their genomes.

Circuits	Inputs	Length of the cell (bits)	No. of cells	Length of the genome (bits)
2-input Ex-OR gate	2	18	3	3 × 18
2-input majority function	3	21	4	4 × 21
2-input majority function	4	23	10	10 × 23
2-to-1 multiplexer circuit	3	21	3	3 × 21

## Data Availability

No Data were used to support this study. However, the findings can be used by researchers for their future work.
